# Tissue sealing versus suture ligation in open canine ovariectomy: Surgical times, intraoperative nociceptive response and frequency of complications

**DOI:** 10.1002/vms3.1012

**Published:** 2022-12-14

**Authors:** Vincenzo Cicirelli, Matteo Burgio, Alice Carbonari, Giovanni M Lacalandra, Giulio G Aiudi

**Affiliations:** ^1^ Department of Veterinary Medicine University of Bari Aldo Moro Bari Italy

**Keywords:** nociceptive response, open canine ovariectomy, surgical complications, surgical times, tissue sealing

## Abstract

**Background:**

In this study, we compared two different techniques currently used for open canine ovariectomy: traditional method utilising absorbable suture and vessel sealing device (ENSEAL^®^ Ethicon Endo‐Surgery, Cincinnati, OH).

**Objectives:**

The aim of this study was to compare the surgical times, intraoperative nociceptive response and the frequency of intraoperative complications in the canine ovariectomy procedure using these two techniques.

**Methods:**

Forty bitches were randomly divided into two groups. The Control Group (C) will use a classic open surgery approach using ligatures with absorbable suture and ovarian resection with a scalpel blade. In the Group E, resection of ovarian structures was performed with ENSEAL^®^ tissue sealer device. For each dog the surgical times, the intraoperative nociceptive response (measuring heart rate, respiratory rate and non‐invasive blood pressure) and the intraoperative complications were measured to compare the effectiveness of the two techniques.

**Results:**

The results of this study showed that the procedures performed using ENSEAL^®^ were faster than the traditional techniques using surgical suture. Instead, the results regarding the nociception and the safety of the two procedures are similar.

**Conclusions:**

The present study shows that the use of ENSEAL^®^ significantly shortened the surgical time. Meanwhile, its use was found to be similarly safe and efficient in terms of intra‐operative nociception, as the classical techniques with absorbable suture. Canine ovariectomy using ENSEAL^®^ device is more practical and faster than the traditional technique; the routine use of this device is considered a useful alternative for the canine neutering.

## Graphical abstract

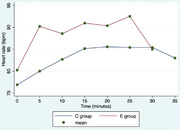

## 1 INTRODUCTION

Ovariectomy (OVE) is one of the most common elective surgical procedures in dogs (Cicirelli et al., 2022b). In the last decades, the canine ovariectomy has been performed using different methods and equipment (Öhlund et al., 2011). Different authors describe the use of suture ligation (Cicirelli et al., 2021), monopolar and bipolar electrocoagulation (Van Goethem et al., 2003, Van Nimwegen and Kirpensteijn, 2007), surgical laser (Van Nimwegen et al., 2005) and ultrasonic devices (Hancock et al., 2005) for the ligation of the ovarian pedicles. The presence of data in literature about the safety, effectiveness and intraoperative nociceptive response of these techniques is limited and scattered (Schwarzkopf et al., 2015). In this study, we compared two different techniques used for open canine ovariectomy: the classical method utilising absorbable suture and the vessel sealing device ENSEAL^®^ for the ligation of the ovarian pedicles. The ENSEAL^®^ system use low voltage, high current, the delivery of which is regulated within the instrument jaws to provide improved vessel compression and sealing with bipolar electro surgery (Schwarzkopf et al., 2015). Indeed, the ENSEAL^®^ proves beneficial for many other surgical indications (e.g. splenectomy, tumours exeresis) (Schwarzkopf et al., 2015). Although it is to be expected that ENSEAL^®^ could improve the procedure, the objective of this study was to compare the surgical times, intraoperative nociceptive response of the patient (considering haemodynamic stability and rescue analgesic administered) (Cicirelli et al., 2021, Cicirelli et al., 2022a, Cicirelli et al., 2021) and the frequency of intraoperative complications (haemorrhages from the ovarian pedicle). To our knowledge, these parameters have not yet been evaluated during canine ovariectomy using ENSEAL^®^. We hypothesise that use of the ENSEAL device will result in decreased surgical time and fewer side effects.

## 2 MATERIALS AND METHODS

### 2.1 1 Animals

This study model is a prospective, randomised clinical trial that involved 40 mixed‐breed owned dogs. Sample size calculation was performed using G*Power for Windows Version 3.1.6 113 (Heinrich Heine Universität Düsseldorf, Germany) (Faul et al., 2007). Previous studies used similar size groups for the assessment of the surgical times, the intraoperative nociceptive response and the intraoperative complications (Cicirelli et al., 2022, Cicirelli et al., 2021, Cicirelli et al., 2021, Cicirelli et al., 2022a). The randomisation process was performed before the trial using StatView statistical software (JMP). They were of good health, without previous pathologies, and were allocated to the very low anaesthetic risk class (ASA 1). Exclusion criteria included obesity (BCS > 4/5) due to the abundance of periovarian fat, aggressiveness, systemic diseases, ovarian and uterine pathologies (excluded using an abdominal ultrasound), bitches in heat and use of drugs in the previous 20 days. The dogs were selected for elective ovariectomy. Informed consent was obtained from pet owners prior to participation in the study. The dogs were randomly divided into two groups. The Control Group (C) used a classic open surgery approach using ligatures with absorbable suture and ovarian resection with a scalpel blade. In the Group E, resection of ovarian structures was performed using ENSEAL^®^ (Ethicon Endo‐Surgery, Cincinnati, OH) tissue sealer device.

### 2.2 Anaesthesia

Standardised surgery and anaesthesia protocols were used for each dog. All dogs were pre‐medicated 20 min before surgery using intramuscular dexmedetomidine (3 μg/kg; Dexdomitor^®^; Vetoquinol Italia SRL, Bertinoro, Italy) and methadone hydrochloride (0.25 mg/kg; Semfortan^®^; Eurovet Animal Health BV, Bladel, the Netherlands) mixed in the same syringe. When the dogs were sedated, a venous catheter was placed in the cephalic vein to start a standard maintenance fluid therapy (NaCl 0.9%, 4 ml/Kg/h) (Cicirelli et al., 2021b), and anaesthesia was induced by intravenous propofol (PropoVet, Zoetis Italia S.r.l.) to effect before intubation. The anaesthesia was maintained with isoflurane (Isoflo^®^, Zoetis Italia S.r.l.) (Cicirelli et al., 2021b). From this point, instrumental monitoring of the following vital parameters was performed (pre‐incision values) and measured every 5 min: heart rate, respiratory rate and non‐invasive blood pressure (GE‐Datex Ohmeda Carestation 620 Anaesthesia Cart, GE‐Datex Ohmeda B 450 Monitor, Milano, Italy) (Cicirelli et al., 2021). In the event of increased of these parameters (>30% compared to pre‐incision values) during the procedure in response to surgical pain in both groups, a bolus of fentanyl was administered intravenously at 2 μg/kg (Fentadon^®^; Eurovet Animal Health BV, Bladel, the Netherlands) as rescue analgesia (Cicirelli et al., 2021).

### 2.3 Surgery

All surgical procedures were performed by the same surgeon and by the same operating team in full compliance with the *lege artis*. Prior to recruiting patients to this study, veterinary surgeons and fellows serving as assistant surgeons had gained familiarity with the ENSEAL^®^ device by performing a minimum of 20 ovariectomy procedures using this instrument (Tapia‐Araya et al., 2015). All procedures were measured by a chronometer from the induction of anaesthesia until the patient awakens. In both groups, the surgeon has performed 2–3 cm incision, 2 cm caudal the umbilical scar to gain entry to the abdomen. The ovary was located using a spay hook and exteriorised (Van Goethem et al., 2006). The suspensory ligament of the ovary was disrupted, when necessary, by manual tearing. The pulling of the ovarian pedicle and the rupture of the suspensory ligament is one of the most painful moments of the procedure. The ligation technique of the ovarian pedicles was performed based on group allocation: for Group C, the ovarian pedicle was clamped securely using Klemmer forceps and circumferential sutures (absorbable monofilament) used to permanently ligate the ovarian blood vessels. Assufil USP 0 (Assut Europe, Italy) was applied between the ovary and the uterine horn, and the vessels ligated. Then, the ovaries were transected (Howe, 2006). In Group E, ovariectomy was performed similarly, but using the Enseal vessel sealing device. The uterine horn was isolated using Kelly forceps, then section and haemostasis of both the uterine vessels was performed by ENSEAL^®^ (Richter et al., 2006). The procedures, of both group, have been concluded with observation for 1 min of any haemorrhages from the abdomen or the remnant ovarian pedicle. The linea alba, subcutaneous tissues and skin were closed routinely. At the end of the procedure, 0.2 mg of Meloxicam^®^ (Metacam, Boehringer Ingelheim Italia S.p.A.) was administered subcutaneously in all animals in this study. Adequate assistance was provided until the righting reflex was observed. The animals were observed for 5 h by the veterinary staff before returning home. The devices were cleaned manually and subsequently sterilised. Sterilisation was performed using a low temperature steriliser (FA 95, Webeco Matachana Group, Barcelona, Spain). The instruments passed a low temperature steaming process with formaldehyde as an active agent.

### 2.4 Data analysis

Compiled forms were entered into a database created using an Excel spreadsheet, and data were analysed using Stata MP17 software. Continuous variables were described as mean (standard deviation [SD]) and range, while categorical variables were described as proportions. The skewness and kurtosis tests were used to evaluate the normality of continuous variables; all continuous variables were normally distributed. The Student's *t*‐test for independent data was used to compare continuous variables between groups, the ANOVA for repeated‐measures test was used to compare continuous variables between groups, and the Fisher's exact test was used to compare proportions. In all tests, a two‐sided *p* value of < 0.05 was considered statistically significant.

## 3 RESULTS

The population consisted of 40 bitches aged 1–4 years and weighing 12–25 kg: 20 (50.0%) in the C group and 20 (50.0%) in the E group.

The outcomes assessed, by group, are described in Table 1.

**TABLE 1:** Outcome by group expressed as mean ± standard deviation (SD) (surgery duration) and as absolute value (rescue analgesia, intraoperative complications)

Variable	C group	E group	Total	*p* Value
Surgery duration	30.2 ± 2.9	22.0 ± 2.9	26.1 ± 5.1	<0.001
Rescue analgesia	1	1	2	1.000
Intraoperative complications	1	1	2	1.000

*Note*: A *p* value < 0.05 was considered significant.

**FIGURE 1:**
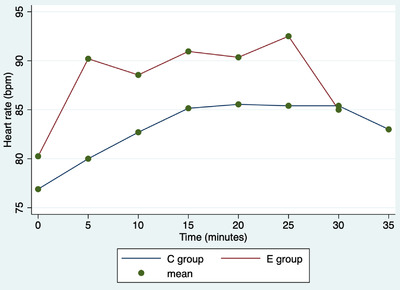
Repeated‐measures ANOVA. Average heart rate (bpm) values in C group (bitches underwent open ovariectomy using standard technique) and E group (bitches underwent open ovariectomy using ENSEAL device) at different times.

**FIGURE 2:**
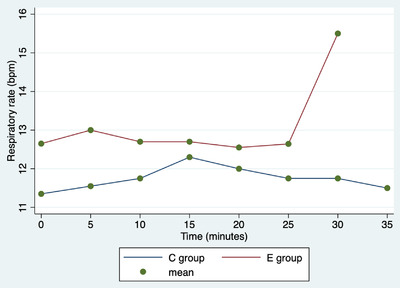
Repeated‐measures ANOVA. Average respiratory rate (bpm) values in C group (bitches underwent open ovariectomy using standard technique) and E group (bitches underwent open ovariectomy using ENSEAL device) at different times.

**FIGURE 3:**
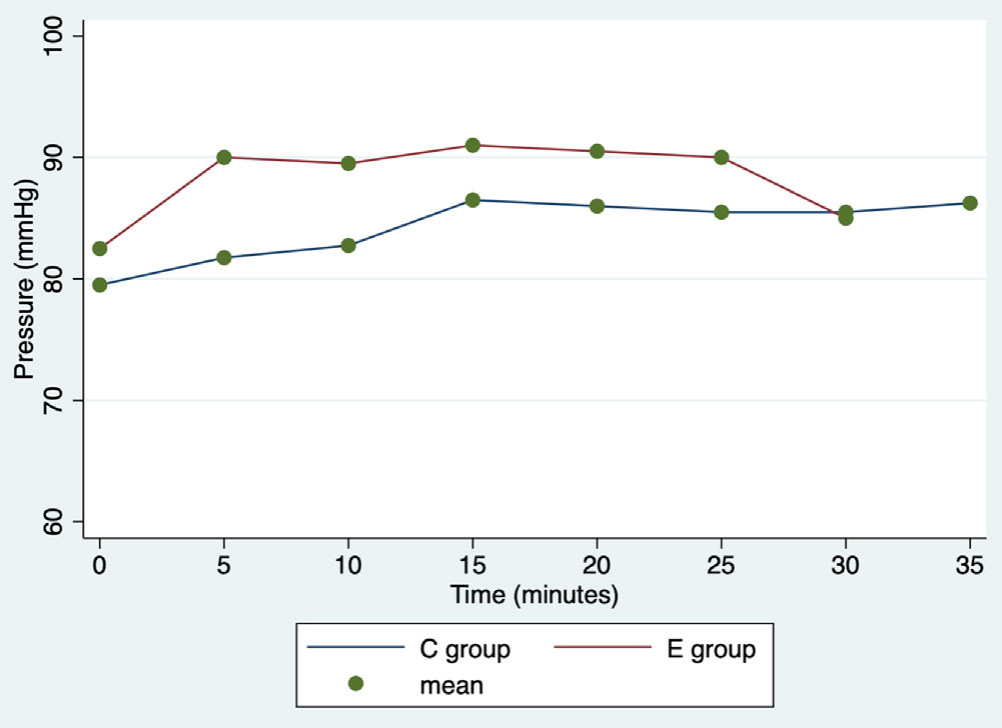
Repeated‐measures ANOVA. Average pressure (mmHg) values in C group (bitches underwent open ovariectomy using standard technique) and E group (bitches underwent open ovariectomy using ENSEAL device) at different times.

In both groups, there was one intraoperative complication, specifically a haemorrhage from the ovarian pedicle. Haemorrhages were promptly managed by ligating the vessels with resorbable suture thread. In both groups, one rescue analgesia was performed, and then, the cardiopulmonary parameters returned to normal. Repeated‐measures ANOVA showed a significant difference among surgery duration and treatment (Graphic 1, coef. = –8.5; 95% CI = –11.3 to –5.6); no further associations with the determinants under analysis are observed (*p* > 0.05). No associations were observed between rescue analgesia and surgery complications and determinants in the analysis (*p* > 0.05). The duration of the procedures is described in Graphic 1. Repeated‐measures ANOVA showed significant differences in cardiac rate among various times (*p* < 0.0001), but not between groups (*p* = 0.190), or in the interaction between time and group (*p* = 0.300; Figure 1). Repeated‐measures ANOVA showed no significant differences in respiratory rate among various times (*p* = 0.901), between groups (*p* = 0.273), or in the interaction between time and group (*p* = 0.601; Figure 2). Repeated‐measures ANOVA showed a significant difference in blood pressure values among the various times (*p* < 0.0001), but not between groups (*p* = 0.161) or in the interaction between time and group (*p* = 0.98; Figure 3).

**GRAPHIC 1:**
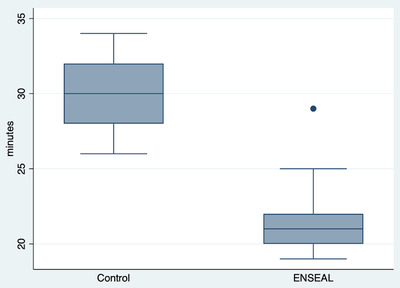
Outcomes assessed in 40 dogs undergoing open ovariectomy with and without use of the ENSEAL^®^ device. A significant difference among surgery duration is showed in 2 groups (C vs. E).

## 4 DISCUSSION

This study did not cause intraoperative serious complications, systemic side effect or death in any of the 40 bitches. No relevant haemodynamic problems were observed during the procedures and no significant haemorrhages were observed during the ovariectomy in both groups, and ovaries were removed without any complications. In fact, the use of ENSEAL^®^ was found to be similarly safe and efficient in terms of intra‐operative nociception, as the classical techniques with absorbable suture. Indeed, an effective ovarian pedicles ligation is essential to prevent any haemorrhages (Berzon, 1979, Person et al., 2008, Pollari et al., 1996, Muraro and White, 2014). The reported causes of ovarian pedicle haemorrhage are vessel laceration during strumming of the suspensory ligament during pedicle handling and manipulations due to improper knot‐tying technique (Adin, 2011). In several studies, intra‐abdominal haemorrhage has been described as the most common complication in canine ovariectomy (Pollari FLBonnett, 1996, Peeters and Kirpensteijn, 2011). Life threatening, intra‐abdominal haemorrhage associated with canine OVE may occur from the ovarian or uterine pedicles or from the suspensory and broad ligaments (Hedlund, 2007, Stone, 2003). In this study, this complication did not occur significantly in any of the two groups. The seal created by the ENSEAL^®^ device is effective, sealing an intrinsic part of the tissue and vessel wall, which cannot be dislodged (Kennedy et al., 1998). Therefore, this study shows that all animals were ovariectomised using an effective anesthetic technique. Meanwhile, its use was found to be similarly safe and efficient in terms of intra‐operative haemostasis as the conventional surgery where, also different vessel‐sealing devices can be used in ovariectomy procedure (Mayhew and Brown, 2007, Culp et al., 2009). In this study, 40 healthy bitches were enrolled. The decision to exclude dogs with BCS >4/5 was made because obese dogs have more periovarian fat, which may affect the standardisation of the study patients (Schwarzkopf et al., 2015). Furthermore, obesity is currently considered a pathology and only healthy dogs were enrolled in this study (ASA 1, 2). The main result of this study was that the use of ENSEAL^®^ significantly shortened the surgical time in canine ovariectomy (30 min in C group vs. 21 min in E group), confirming our hypothesis. The difference in procedure duration between two groups was averaged 9 min. A period of 9 min over a routine procedure lasting an average of 30 min reduces surgical time by a third. Reducing surgical time is useful for reducing the occurrence of problems such as surgery‐related infections, loss of patient temperature and adverse effects of anaesthetic agents used for anaesthesia (Van Goethem et al., 2006). Previous studies evaluated different techniques for ovarian pedicle haemostasis in canine ovariectomy (Öhlund et al., 2011, Van Nimwegen et al., 2005, Howe, 2006), among which tissue sealing device such as ENSEAL^®^ has successfully been used. Indeed ENSEAL^®^ do reliably seal large vessels (as ovarian vessels), relying on a feedback‐controlled response system that automatically discontinues the electrocoagulation process when the tissue is adequately sealed (Richter et al., 2006). The use of ENSEAL^®^ reduces thermal necrosis and collateral damage when compared with traditional systems in which the amount of energy is subjectively controlled by the operator (Dupre et al., 2009, Santini et al., 2006, Pastore et al., 2013). However, there are some disadvantages such as the cost of equipment and the need for specialised training (Gower and Mayhew, 2008, Leitch et al., 2012). Potential disadvantages of the ovariectomy procedure using ENSEAL include equipment expense, need for specialised training and need for additional assistants. The evaluation of pain stemmed from a general trend towards increasing attention on intraoperative pain during surgical procedure in dog (Cicirelli et al., 2022). Pain management in veterinary patients is a crucial component of appropriate patient care (Cicirelli et al., 2022a). In fact, acute intraoperative pain is of great interest due to potential risk of becoming chronic if not treated properly. In this study, intraoperative nociception was measured by assessing increases in cardiopulmonary parameters, which then led to administration of rescue analgesia. Indeed, a bolus of Fentanyl was administered in the event of an increased heart rate, respiratory rate or blood pressure (>30% compared to pre‐incision values) during the procedure in response to surgical pain in both groups. The correlation between rescue analgesia and intraoperative nociception is commonly used as indicators in these types of studies (Weary et al., 2006). In fact, when the sympathoadrenal system is stimulated by a nociceptive stimulus, heart rate, respiratory rate and blood pressure increase. For this reason, supportive analgesia is necessary (Cicirelli et al., 2022). In our result, only 1 animal per group required intraoperative rescue analgesia. This confirms that a good anaesthetic/analgesic protocol was used in both groups and the use of ENSEAL^®^ does not affect the intraoperative analgesia used. The limitation of this study is that it cannot be blinded to the type of experiment. More information can be gathered on the possible bias employed by the members of the surgical team.

## 5 CONCLUSION

This study demonstrated that the canine ovariectomy using ENSEAL^®^ is faster than the traditional techniques using surgical suture, without affecting the safety and nociception of neutering. Indeed, routine use of the ENSEAL^®^ is considered desirable for canine ovariectomy. The clinical implication of the current study may be a more widespread use of ENSEAL^®^ for canine ovariectomy among practitioners.

## AUTHOR CONTRIBUTIONS

Vincenzo Cicirelli: conceptualisation, writing – original draft. Matteo Burgio: investigation, writing – original draft. Alice Carbonari: writing – review & editing. Giovanni Michele Lacalandra: project administration, resources. Giulio Aiudi: methodology, supervision.

## CONFLICT OF INTEREST

All authors collaborated in the same way for the drafting of this work. Prof Aiudi supervised the teamwork. The authors declare that they have no competing interests.

## FUNDING

This research received no external funding.

## ETHICS STATEMENT

The present study was performed in accordance with the ethical guidelines of the Animal Welfare Committee, and Institutional Review Board approval of the study was obtained (approval number: 30/21 [12/11/2021]). Animal procedures were performed following Directive 2010/63/EU of the European Parliament (Italian DL 26/2014).

### PEER REVIEW

The peer review history for this article is available at https://publons.com/publon/10.1002/vms3.1012.

## Data Availability

The data presented in this study are available on request from the corresponding author.
